# Reducing and Uniforming the Co_3_*O*_4_ Particle Size by Sulfonated Graphenal Polymers for Electrochemical Applications

**DOI:** 10.1186/s11671-017-1953-8

**Published:** 2017-03-04

**Authors:** Xin Zhang, Xubo Liu, Sha Zeng, Jianhui Fang, Chuanling Men, Xiaohua Zhang, Qingwen Li

**Affiliations:** 1 Department of Chemistry, College of Sciences, Shanghai University, Shanghai, 200444 China; 2 Suzhou Institute of Nano-Tech and Nano-Bionics, Chinese Academy of Sciences, Suzhou, 215123 China; 3 School of Energy and Power Engineering, University of Shanghai for Science and Technology, Shanghai, 200093 China

**Keywords:** Sulfonated graphenal polymer, Cobalt oxide, Hydrothermal growth, Electrochemical

## Abstract

A novel two-dimensional (2D) nanomaterial, namely sulfonated graphenal polymer (SGP), is used to tune the hydrothermal growth of Co_3_O_4_ nanoparticles. SGP provides abundant nucleation sites to grow Co_3_O_4_ nanoparticles and effectively reduces the particle size and dimension. As a result, with considering the improved size uniformity of Co_3_O_4_ and the tight wrapping of SGP around Co_3_O_4_ as well, the Co_3_O_4_/SGP hybrid electrode exhibits a high specific electrochemical capacitance of 234.28 F/g at a current density of 0.2 A/g, 237% higher than that of the pure Co_3_O_4_ electrode. By using the hybrid as the anode of an all-solid-state asymmetric supercapacitor, the capacitance can be well maintained up to 93% after 5000 cycles even at 2 A/g.

## Background

Owing to their high power density, cycle efficiency, and charge/discharge rate, supercapacitors are considered as promising candidates in energy storage applications [1–4]. There are two types of supercapacitors according to their different charge storage mechanisms, namely electric double-layer capacitors and pseudocapacitors [5]. Electric double-layer capacitor stores energy by interfacial charge separation between its electrode and the electrolyte, while pseudocapacitor realizes the charge storage mainly through the pseudo-faradaic reactions [6]. The former usually has a high performance in power density and the latter is preferred to approach a high energy density. To further improve the energy density, and specific capacitance as well, transition metal oxides, e.g., Co_3_O_4_, MnO_2_, NiO, and RuO_2_, are widely used in the electrodes of supercapacitors [7–10]. In general, these transition metal oxides should be small sized (towards a high surface area), uniformly distributed, and highly crystallized. Obviously, the synthesis and assembly of these particles are of great importance for the electrode performances, especially for the energy density and rate of capacitance [11].

Hydrothermal process is a common way to grow metal oxide nanoparticles [12]. To control the particle size, crystallinity, and particle morphology, the introduction of nanoplatelets has been proved as an efficient strategy [13–16]. For example, graphene oxide (GO) has exhibited exciting effects as it can provide rich active sites to anchor the nanoparticles, prevent their agglomeration, and avoid the overgrowth. This is owing to its two-dimensional (2D) nanostructure, abundant functional groups, and high specific surface area [15, 16]. As a result, needle-like Co_3_O_4_ nanoparticles with a size of 10–50 nm or cross-like NiO nanoflakes with a thickness of ∼15 nm were grown on GO [17, 18].

In this study, we report a facile way to precisely control the size and distribution of Co_3_O_4_ nanoparticles on a new type of 2D nanostructure, namely sulfonated graphenal polymer (SGP), by a hydrothermal process. On the one hand, the abundant sulfonic acid groups of SGP make it superhydrophilic and thus allow efficient adsorption of metal cations [19]. Therefore, there exist rich anchor sites for the growth of Co_3_O_4_. On the other hand, the polymeric characteristics of SGP [20], due to its irregular or branched 2D planar structure, result in high flexibility and thus tight wrapping of SGP around the Co_3_O_4_ nanoparticles.The Co_3_O_4_/SGP hybrid electrode can exhibit a high specific capacitance of 234.28 F/g at a current density of 0.2 A/g, which was 237% higher than that of the pure Co_3_O_4_ electrode (69.5 F/g). By using the Co_3_O_4_/SGP electrode as the anode of an all-solid-state asymmetric supercapacitor, a high reversibility was achieved due to the reduced and uniformized particle size and intimate contact between the Co_3_O_4_/SGP hybrid with the conducting fillers. After 5000 cycles even at 2 A/g, the capacitance was still maintained up to 93%. As compared to GO, this study demonstrates an interesting potential of SGP in the electrochemical applications.

## Methods

### Materials Synthesis

The SGP nanostructures in this study were purchased from Suzhou Graphene-Tech Co., Ltd. and were used as received. They were synthesized by a “bottom-up” strategy [20] where the reaction between polyethylene and fluorosulfuric acid were conducted [21]. After the fluorosulfuric acid (concentration over 99%) was heated up to 70–80 °C, polyethylene was added to trigger the reaction. The polymer-to-acid weight ratio was within 1:10 to 1:15. After 10 min, the reaction product was separated by water to obtain SGP dispersions, which can be further dried to obtain SGP powders. In this study, the as-received SGP powder was dispersed in deionized water to obtain a 0.1 wt% SGP solution.

For the Co_3_O_4_ growth, 1 mmol cobalt acetate (Co(CH_3_COO)_2_ ·4H_2_O or Co(OAc)_2_ ·4H_2_O) was dissolved in 45 ml water, mixed with 5 ml 0.2 M aqueous NaOH solution, and then stirred for 5 min. The solution was transferred into Teflon-lined stainless steel autoclave and then maintained at 120 °C for 12 h to start the Co_3_O_4_ growth. After the reaction, the solution was cooled naturally to room temperature and then dried at 80 °C for 12 h. The SGP-assisted Co_3_O_4_ growth had the same treatment sequence, except additional 5 ml 0.1 wt% SGP solution was added into the mixture (the total volume was still 50 ml just by dissolving the same amount of Co(OAc)_2_ into 40 ml water). In order to study the effect of the solution basicity, the similar Co_3_O_4_ growth was also performed in an NH_3_ solution. Here, NaOH was replaced by 9 ml NH_3_ ·H_2_O without tuning the amounts for the other compounds (the total volume was also fixed to 50 ml). By using these two different growths, Co_3_O_4_/SGP hybrid or composite powders were obtained.

### Electrode and Supercapacitor Preparations

Five milligrams of Co_3_O_4_ or Co_3_O_4_/SGP powder was added to ethanol, and then blended with 0.5 mg multiwall carbon nanotubes (MWCNTs) which acted as the conductive agent. The mixture was dried at 80 °C for 1 h and then packaged with a 1-cm ^2^ Ni foam. The foam was dried at 110 °C for 12 h under a vacuum oven, and then pressed under 10 MPa to form a working electrode.

To better evaluate the electrochemical performance, a supercapacitor was made by assembling the Co_3_O_4_ electrode as the anode, active carbon as the cathode, and poly(vinyl alcohol) (PVA)/KOH as the separator. The gravimetric specific capacitance for the whole supercapacitor cell was calculated from the galvanostatic charge/discharge (GCD) curve by considering the total mass of Co_3_O_4_, SGP, and CNT, namely, the mass increase of the Ni foam.

### Electrochemical Measurements

The electrochemical performance of electrode was measured using a three-electrode test in a 6 M KOH solution at room temperature, with a Chenhua CHI-660C electromechanical workstation. A platinum mesh and a saturated Hg/HgO electrode were used as the counter and reference electrodes, respectively. Cyclic voltammetry (CV), GCD, and electrochemical impedance spectroscopy (EIS) were carried out using conventional three-electrode configurations at a potential range of 0–0.4 V or in a frequency range of 100 kHz to 0.01 Hz. The specific capacitance (*C*_*s*_) was calculated from the GCD data by *C*_*s*_=*It*/*m* *Δ* *V*, where *I* is the applied current, *t* the time for the discharge process, *m* the mass of the active material, and *Δ* *V* the potential range [22, 23].

### Characterizations

The Co_3_O_4_ structure was characterized by a Quanta 400 FEG field emission scanning electron microscope (SEM) equipped with an Apollo 40 SDD energy-dispersive X-ray spectroscope (EDS) for element determination and by an FEI Tecnai G2 F20 S-Twin transmission electron microscope (TEM). Lateral dimensions and thickness of SGP nanosheets were characterized using tapping-mode atomic force microscope (AFM; Bruker Instruments Dimension Icon) with a silicon-tip cantilever (40 N/m). The Fourier transform infrared spectroscopy (FTIR) (500–4000 cm ^−1^) was measured using a Nicolet 6700 FTIR spectrometer. The surface area was measured by the Brunauer-Emmett-Teller (BET) method with a Micromeritics ASAP 2010 sorptometer. The crystal structure was examined by a Bruker AXS D8 Advance X-ray diffraction (XRD) system.

## Results and Discussion

### Structure of SGP

**Fig. 1:**
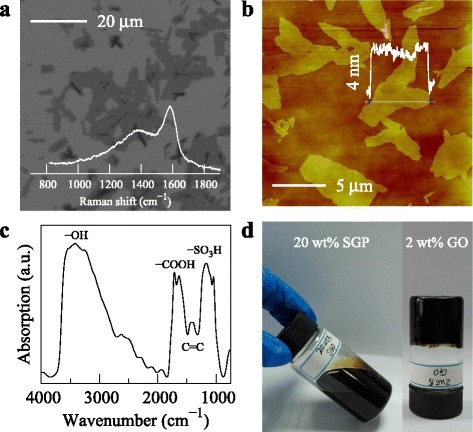
**a, b** SEM and AFM images of SGP nanosheets with insets showing the Raman spectrum and film thickness. **c** FTIR spectrum shows high fractions of −SO_3_H, −COOH, C=C, and −OH groups. **d** Optical images of a 20 wt% SGP dispersion (still a liquid) and 2 wt% GO dispersion (gel like)

As a new type of graphenal polymer [20], SGP possessed common features such as large specific surface area, good physical and chemical stability, flexibility, and adhesiveness. So far, the first study on SGP was the application in the electrolyte separator for all-solid-state supercapacitors where SGP nanosheets were self-assembled into a highly porous film with the aid of intermolecular adhesion by PVA [24]. To show the nanostructure of SGP, SEM and AFM were performed, see Fig. 1 a, b. SGP showed a 2D planar structure while the 2D plane was quite irregular as porous and branched structures were widely observed (Fig. 1 a). This was a result of the bottom-up synthesis procedure [20]. The SGP nanosheets were about 4–5 layered according to the thickness of ∼4 nm (Fig. 1 b). The Raman spectrum showed two peaks centered at 1585 and 1382 cm ^−1^ which were typically the G- and D-peaks for the carbon sp ^2^ structure (Fig. 1 a, inset).

Further, the sulfonic acid group (−SO_3_H), carboxyl group (−COOH), and hydroxyl group (−OH) were detected as strong absorption peaks with FTIR, as shown in Fig. 1 c. Besides the wide absorption band for −OH centered around 3200–3550 cm ^−1^, the absorption bands at 1043 and 1171 cm ^−1^ were due to the SO _2_ symmetric and asymmetric stretching, and the absorption between 1650–1720 cm ^−1^ was related with −COOH. Based on these structural characteristics, such 2D nanostructure was named as sulfonated graphenal polymer. Furthermore, the abundant sulfonic acid and carboxyl groups resulted in superhydrophilicity for SGP, as these molecules can be dispersed in water at high concentrations even up to 26 wt%. As shown in Fig. 1 d, the 20 wt% SGP dispersion still showed a high fluidity while the 2 wt% GO dispersion nearly became a hydrogel.

### SGP-assisted Co_3_O_4_ Growth

Co_3_O_4_ nanoparticles were fabricated with a hydrothermal approach by using Co(OAc)_2_ as the source material. Under a basic environment (0.02 M NaOH solution), Co(OAc)_2_ converted into Co_3_O_4_ after several hours, according to the following reactions [14, 25, 26]:

1 $$\begin{array}{@{}rcl@{}} \text{Co}^{2+} + 2\text{OH}^{-} &\longrightarrow& \text{Co}(\text{OH})_{2}, \end{array} $$

2 $$\begin{array}{@{}rcl@{}} \text{Co}(\text{OH})_{2} +\mathrm{O}_{2} &\longrightarrow& \text{Co}_{3}\mathrm{O}_{4} + \mathrm{H}_{2}\mathrm{O}. \end{array} $$

**Fig. 2:**
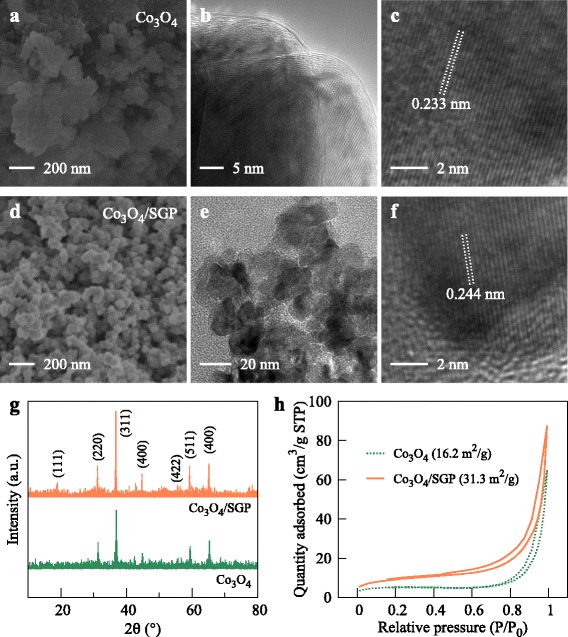
**a**–**c** SEM and TEM images showing that the Co_3_O_4_ particles were 50–200 nm sized and the interplanar distance of 0.233 nm corresponded to the stacking of (311) planes. **d**–**f** SGP reduced the particle size down to sub-20 nm, while the similar (311) stacking was observed with an interplanar distance of 0.244 nm. **g**, **h** XRD patterns and N_2_ adsorption-desorption isotherms for the Co_3_O_4_ and Co_3_O_4_/SGP powders

SGP can strongly influence the Co_3_O_4_ growth. Without SGP, the particle size was quite diverse, ranging from 50 to 200 nm (Fig. 2 a and b). The large-sized Co_3_O_4_ nanoparticles were the agglomeration of small-sized particles due to the lack of nucleation sites [27]. On the contrary, the presence of SGP can provide sufficiently more nucleation sites to anchor Co(OH)_2_ clusters for the direct Co_3_O_4_ growth on SGP, owing to the abundant −OH groups [28]. These groups can also reduce the divergence in growth rate at different sites and hinder the overgrowth due to the space limit. As a result, the SGP-assisted growth showed an improved uniformity in particle size, see Fig. 2 d, e. In general, even after a 12-h growth, the Co_3_O_4_ particles on SGP were still about 20–30 nm in diameter, while the free growth just in 3 h could cause a particle size over 100 nm.

The reduced particle size did not affect the crystallinity. Figure 2 c, f compares the TEM results for the free and SGP-assisted growths. Both structures showed an inter-planar spacing of 0.233–0.244 nm, by averaging the length of 20 lattices, corresponding to the (311) plane of fcc Co_3_O_4_. XRD measurements (Fig. 2 g) also showed diffraction peaks at 18.9°, 31.1°, 36.8°, 44.7°, 55.6°, 59.3°, and 65.1°, which were assigned to (111), (220), (311), (400), (422), (511), and (440) crystal planes. For both growths, the (311) diffraction peak was the most strongest one, in agreement with the TEM characterization.

The reduced particle size increased significantly the specific surface area. Figure 2 h shows a comparison of the BET surface area. The presence of SGP increased the specific surface area from 16.2 m ^2^/g for the free growth up to 31.3 m ^2^/g. The improved surface area could increase the contact area between the electrode and electrolyte, provide more surface reaction sites, and thus benefit the electrochemical performance.

### Electrode Performance

The electrochemical properties of the Co_3_O_4_ and Co_3_O_4_/SGP electrodes were investigated with a three-electrode test in a 6 M KOH solution. The electrodes were prepared in a conventional way by using MWCNTs as conductive additives. 0.5 mg MWCNTs were blended with 5 mg Co_3_O_4_ or Co_3_O_4_/SGP powders, and then packaged inside 1-cm ^2^ Ni foam. In electrochemical measurement, the potential window was 0–0.4 V against the Hg/HgO reference electrode [8, 29]. The redox reactions at the Co_3_O_4_ electrode can be described by the following sequences [16]:

3 $$\begin{array}{*{20}l} &\text{Co}_{3}\mathrm{O}_{4} + \mathrm{H}_{2}\mathrm{O} + \text{OH}^{-} \longleftrightarrow 3\;\text{CoOOH} + \mathrm{e}^{-}, \end{array} $$

4 $$\begin{array}{*{20}l} &\text{CoOOH} + \text{OH}^{-} \longleftrightarrow \text{CoO}_{2} + \mathrm{H}_{2}\mathrm{O} + \mathrm{e}^{-}. \end{array} $$

**Fig. 3:**
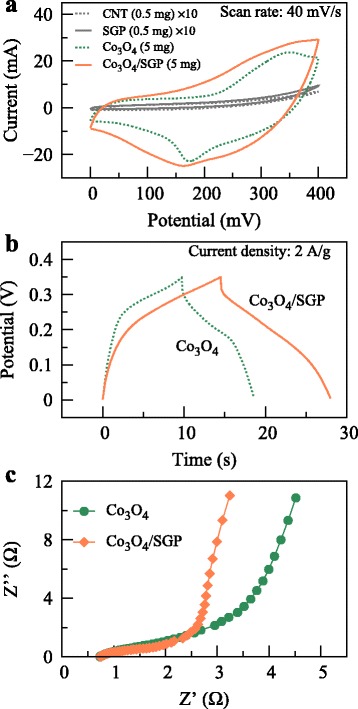
**a** CV curves at a scan rate of 40 mV/s for the Co_3_O_4_ and Co_3_O_4_/SGP hybrid electrodes. The mass of activate materials was 5 mg. For a comparison, the CV curves for SGP and CNT (mass 0.5 mg) were provided, with a magnification factor of 10. **b** GCD behaviors at a current density of 2 A/g. **c** EIS spectra measured at a frequency range of 100 kHz to 0.01 Hz

The reduced particle size and increased surface area of Co_3_O_4_ benefited the electrochemical properties. As compared to the free-grown Co_3_O_4_, the Co_3_O_4_/SGP hybrid electrode exhibited enhanced CV and GCD performances (Fig. 3 a, b). The specific capacitance of the Co_3_O_4_/SGP hybrid electrode was up to 100.3 F/g at a current density of 0.2 A/g, which was 144% higher than that of the Co_3_O_4_ electrode (69.5 F/g at 0.2 A/g). The charge/discharge time at 2 A/g also increased from 19 to 28 s after the SGP-assisted growth. Notice that both SGP and CNT did not contribute to the electrochemical activity by themselves as they are much less active than Co_3_O_4_. In Fig. 3 a, the CV curves for SGP and CNT are also provided for comparison, where these carbon materials were packed into Ni foams at a mass of 0.5 mg.

To better understand the improved performance of the Co_3_O_4_/SGP electrode, EIS was employed to investigate the reaction kinetics. The Nyquist plots of different samples, which were characterized by a semicircle in the high-frequency region and a straight line in the low-frequency region, are shown in Fig. 3 c. In general, the bigger diameter of the semicircle, the bigger charge transfer resistance between the electrode and electrolyte [30]. Obviously, the hybrid sample showed a lower charge transfer resistance, corresponding to the improved electric conductivity.

### Effect of Solution Basicity

NaOH is a strong base and can induce rapid growth of Co_3_O_4_, as the highly polar OH^−^ groups can easily coordinate with Co ^2+^. As a result, the as-produced Co_3_O_4_ nanoparticles were spherical in shape even under the SGP-assisted growth, see Fig. 2 d–f. In order to suspend the growth rate and thus restrict the particle shape or size, a less basic solution, a 2.36 M NH_3_ solution, was used to produce the Co_3_O_4_/SGP hybrids. The synthetic processes were based on the relatively weak coordination between Co^2+^ and NH_3_, as described by the following reactions [31–33]:

5 $$\begin{array}{*{20}l} &{}\text{Co}^{2+} + 6\,\text{NH}_{3} \longrightarrow \left[\text{Co}(\text{NH}_{3})_{6}\right]^{2+}, \end{array} $$

6 $$\begin{array}{*{20}l} &{}4\left[\text{Co}\left(\text{NH}_{3}\right)_{6}\right]^{2+} + \mathrm{O}_{2} + 2\,\mathrm{H}_{2}\mathrm{O} \longrightarrow 4\left[\text{Co}\left(\text{NH}_{3}\right)_{6}\right]^{3+}\\ &\qquad\qquad\qquad\;\, + 4\,\text{OH}^{-}. \end{array} $$

Then, these coordinate ions decomposed at a certain temperature (usually via a hydrothermal process), and finally, Co_3_O_4_ nanomaterials were obtained [31].

**Fig. 4:**
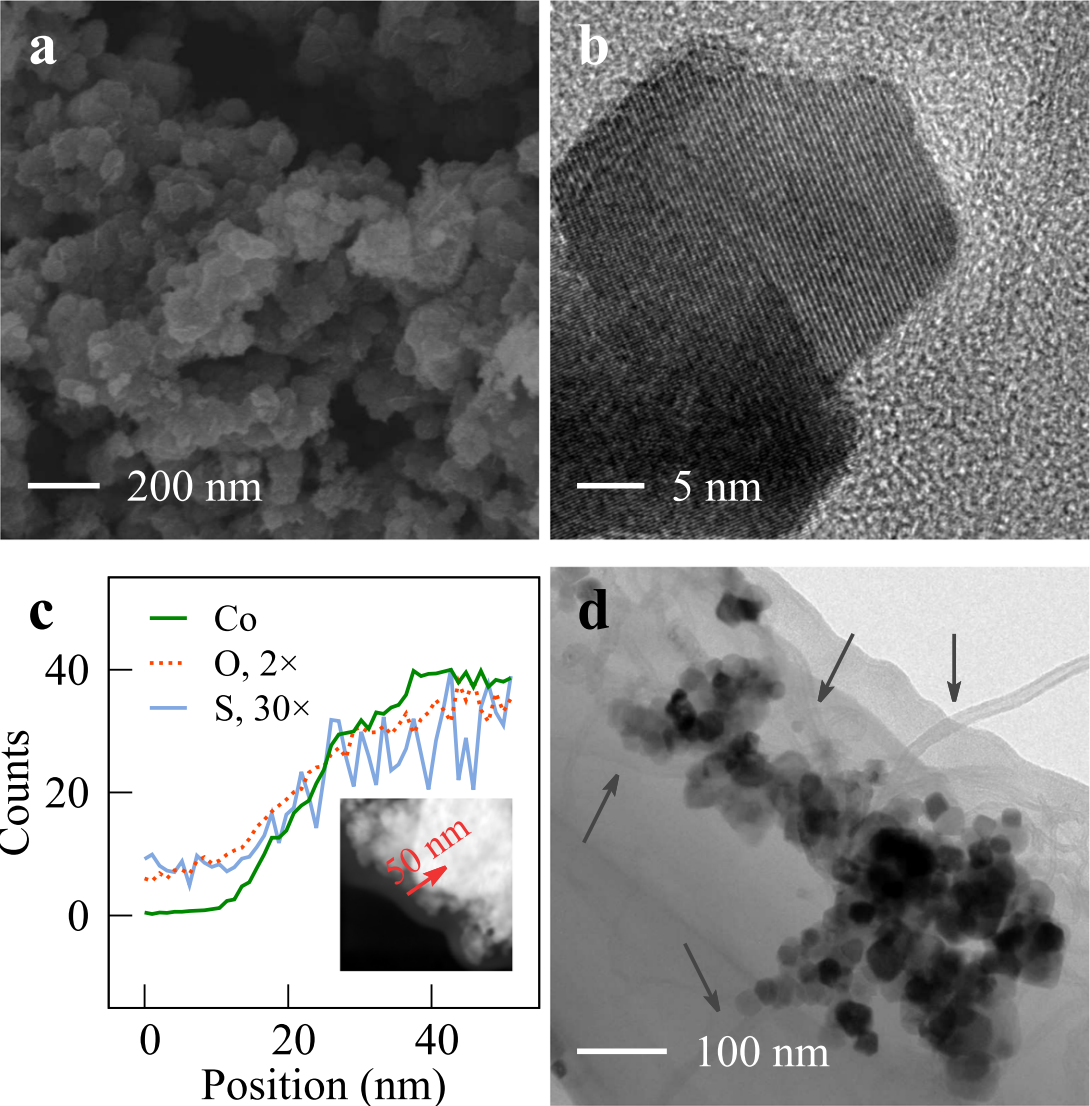
**a** The Co_3_O_4_/SGP nanoparticles grown in NH_3_ solutions were agglomerations of 2D nanoplatelets. **b** The Co_3_O_4_ nanoplatelets had a lateral dimension of 25–30 nm. **c** EDS characterization showed that the Co_3_O_4_ nanoparticles were tightly wrapped by SGP. **d** After blending, the Co_3_O_4_ nanoplatelets had an intimate contact with MWCNTs, labeled by *arrows*

Figure 4 a shows the agglomeration morphology of Co_3_O_4_ nanoparticles obtained in the NH_3_ solution. As compared to Fig. 2 d, the particle did not have a smooth surface but looked like assemblies of low-dimensional nano-structures. TEM characterization showed that 2D Co_3_O_4_ nanoplatelets with lateral size of 25–30 nm were grown in the NH_3_ solution (Fig. 4 b). Clearly, the particle size was well controlled both in the particle size due to the presence of growth substrate of SGP and in the particle dimension due to the decreased basicity.

Nevertheless, a general structure was obtained despite the different basic solutions. By tracing from an empty place to the surface of a Co_3_O_4_ nanoparticle, the element counting of Co, O, and S increased simultaneously at the particle surface, see the EDS curve shown in Fig. 4 c. This means that the Co_3_O_4_ nanoparticles were tightly wrapped by SGP, clearly due to its irregular and flexible 2D structure. Notice that, from SEM or TEM images in Figs. 2 and 4, it was difficult to observe any planar nanostructures as shown in Fig. 1. This was another important observation to show the high flexibility of SGP. Such tight wrapping can reduce the charge transfer distance from the surrounding solution to the inside of the nanoparticle, and thus is important for the electrochemical performance. Furthermore, these Co_3_O_4_/SGP hybrid nanoparticles formed intimate contact with MWCNTs after the blending to prepare electrodes, see the TEM image in Fig. 4 d. This also indicated that the small-sized and low-dimensional Co_3_O_4_ nanoplatelets could exhibit superior electrochemical performances.

Notice that, the SGP-assisted growth can be directly compared with the Co_3_O_4_ growth on GO [32], by using the same source material (Co(OAc)_2_) and basic solution (NH_3_ ·H_2_O). Due to the reduced basicity, both growths produced Co_3_O_4_ nanoparticles with a particle size no larger than 25 nm, attributed to the coordination between Co^2+^ and NH_3_ in reducing the Co_3_O_4_ particle size [31, 32]. However, clear difference could be observed at the particle dimension. The less functionalization level of GO, as compared to SGP, still causes the formation of 3D Co_3_O_4_ nanospheres, different from the SGP-induced dimension reduction of Co_3_O_4_ nanoplatelets.

**Fig. 5:**
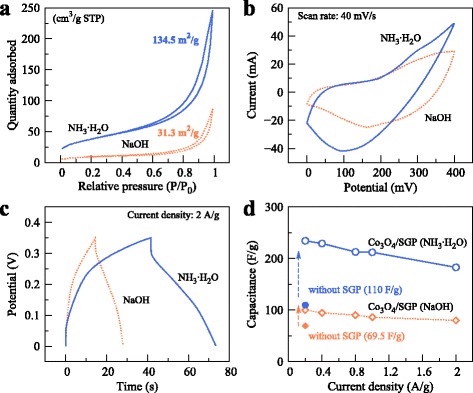
**a** N_2_ adsorption-desorption isotherms for the Co_3_O_4_ nanoparticles grown in the NaOH and NH_3_ solutions. **b**, **c** Comparisons in CV and GCD for different Co_3_O_4_ growths. **d** Specific capacitances at current densities ranging from 0.2 to 2 A/g. For a comprehensive comparison, the capacitances at 0.2 A/g for the free growth in different solutions were also plotted

The reduction in particle dimension could greatly benefit the specific surface area. Figure 5 a compares the BET measurements of the Co_3_O_4_ nanoparticles obtained from the two solutions. Surprisingly, the dimension reduction increased the specific surface area significantly from 31.3 to 134.5 m ^2^/g. Based on such advantage, the electrode performance was also improved remarkably by NH_3_. Under a scan rate of 40 mV/s, the internal area of CV curve for Co_3_O_4_/SGP composite grown in NH_3_ was apparently larger than that of Co_3_O_4_/SGP composite grown in NaOH (Fig. 5 b), implying that the specific capacitance could be improved remarkably by NH_3_. The current intensity at oxidation peaks of CV increased from 25 to 30 mA at a voltage of ∼350 mV, and the redox peak also became broader. By comparing Fig. 3 a and Fig. 5 b, one can find that the CV curve became more rectangular and the redox peaks became much wider, possibly due to the enhanced charge transfers from the dimension reduction. Figure 5 c further shows that the charge/discharge time at 2 A/g increased from 28 to 32 s by changing the basic environment from NaOH to NH_3_. As a result, the specific capacitance of the Co_3_O_4_/SGP (obtained by NH_3_) hybrid electrode was up to 234.28 F/g at a current density of 0.2 A/g (Fig. 5 d), which was 134% higher as compared to the SGP-assisted growth in NaOH (100.3 F/g), and was 3.37 and 2.13 times those of the pure Co_3_O_4_ electrodes obtained from NH_3_ (69.5 F/g) and NaOH (110 F/g), respectively.

Notice that, the electrode capacitance was not as high as up to about 500–1000 F/g in many recent literature [8, 12, 14, 16]. The possible reasons could be the un-intimate contact between the activate material with Ni foam, the low electrical conductivity of CNT, and the un-uniform distribution of Co_3_O_4_ with CNT, due to the physical blending. This was similarly reported in some other literature where the capacitance was only up to about 150–270 F/g [17, 34, 35]. Nevertheless, as all the electrodes were prepared under the same method, the present study could still show the exciting advantage of SGP in improving the deposition of metal oxides.

### Application in Supercapacitors

To further investigate the Co_3_O_4_/SGP electrode performance, the hybrid was assembled with active carbon and PVA/KOH electrolyte to an all-solid-state asymmetric supercapacitor, where the Co_3_O_4_/SGP, active carbon, and PVA/KOH acted as the anode, cathode, and separator, respectively. The two electrodes were integrated with Ni foams for a better current collection. The gravimetric specific capacitance for the whole supercapacitor cell was calculated from the GCD curve by considering the total mass of the two electrodes.

**Fig. 6:**
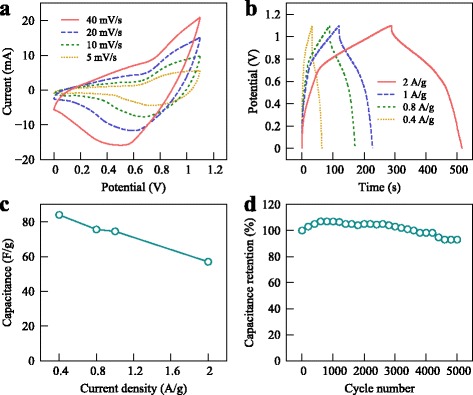
**a** CV curves for an asymmetric supercapacitor with the Co_3_O_4_/SGP acting as the anode. **b**, **c** GCD curves and specific capacitances at different current densities. **d** Cycling performance at a current density of 1 A/g

In the supercapacitor, the Co_3_O_4_/SGP electrode exhibited explicit redox peaks of pseudocapacitor and excellent cycle stability. With increasing the scan rate from 5 to 40 mV/s, the current intensity at oxidation peaks of CV increased correspondingly from 2.5 to 9.7 mA (Fig. 6 a). The GCD curves of the Co_3_O_4_/SGP at different current densities showed the Faradaic characteristics, in agreement with the CV curves (Fig. 6 b). The specific capacitance was 84.0, 75.6, 74.5, and 57.0 F/g at current densities of 0.4, 0.8, 1, and 2 A/g, respectively, reflecting the common performance in the three-electrode system (Fig. 6 c).

Furthermore, the cycle charge/discharge test was employed at a current density of 1 A/g, as shown in Fig. 6 d. Owing to the small-sized Co_3_O_4_ and the SGP wrapping, the device demonstrated an excellent stability, remaining 93% of its initial capacitance even after 5000 charge/discharge cycles. Notice that the specific capacitance slightly grew up in the first 500 cycles. This could be a result of electrochemical activation due to the increase in contact area between the electrode and the electrolyte during the cycling [36]. As compared to the previous reports [17], where ∼70% capacitance was maintained after 4000 cycles, the present results obviously demonstrate an exciting potential for the application in energy storage devices.

## Conclusions

SGP-wrapped Co_3_O_4_ nanoparticles with uniform particle size were synthesized with a hydrothermal process. As a result of the reduced particle size and wrapping assembly, the Co_3_O_4_/SGP hybrid exhibited superior electrochemical performance due to the increased BET surface area and the improved interfacial electrical conductivity. By tuning the basic solution from NaOH to NH_3_, the particle dimension was further reduced, as reflected by the production of Co_3_O_4_ nanoplatelets with lateral size of 25–30 nm. For the electrochemical performance, excellent stability was observed both in the improved rate capacity of single electrode and the capacitance maintenance of supercapacitor. This study provides a new approach to produce uniformly sized Co_3_O_4_ nanoparticles for the application in energy storage devices.

## Abbreviations

AFM: Atomic force microscopy
BET: Brunauer-Emmett-Teller
CV: Cyclic voltammetry
EIS: Electrochemical impedance spectroscopy
EDS: Energy-dispersive X-ray spectroscopy
FTIR: Fourier transform infrared spectroscopy
GO: Graphene oxide
GCD: Galvanostatic charge/discharge
MWCNTs: Multiwall carbon nanotubes
PVA: Poly(vinyl alcohol)
SEM: Scanning electron microscopy
SGP: Sulfonated graphenal polymer
TEM: Transmission electron microscopy
XRD: X-ray diffraction

## References

[1] Simon P, Gogotsi Y (2008). Materials for electrochemical capacitors. Nat Mater.

[2] Dunn B, Kamath H, Tarascon JM (2011). Electrical energy storage for the grid: a battery of choices. Science.

[3] El-Kady MF, Kaner RB (2013). Scalable fabrication of high-power graphene micro-supercapacitors for flexible and on-chip energy storage. Nat Commun.

[4] Miller JR, Simon P (2008). Electrochemical capacitors for energy management. Science.

[5] Wang H, Liang Y, Mirfakhrai T, Chen Z, Casalongue HS, Dai H (2011). Advanced asymmetrical supercapacitors based on graphene hybrid materials. Nano Res.

[6] Zhi M, Xiang C, Li J, Li M, Wu N (2013). Nanostructured carbon-metal oxide composite electrodes for supercapacitors: a review. Nanoscale.

[7] Chen H, Zhou S, Wu L (2014). Porous nickel hydroxide-manganese dioxide-reduced graphene oxide ternary hybrid spheres as excellent supercapacitor electrode materials. ACS Appl Mater Interfaces.

[8] Dong XC, Xu H, Wang XW, Huang YX, Chan-Park MB, Zhang H (2012). 3D graphene-cobalt oxide electrode for high-performance supercapacitor and enzymeless glucose detection. ACS Nano.

[9] Luan F, Wang G, Ling Y, Lu X, Wang H, Tong Y (2013). High energy density asymmetric supercapacitors with a nickel oxide nanoflake cathode and a 3D reduced graphene oxide anode. Nanoscale.

[10] Susanti D, Tsai DS, Huang YS, Korotcov A, Chung WH (2007). Structures and electrochemical capacitive properties of RuO_2_ vertical nanorods encased in hydrous RuO_2_. J Phys Chem C.

[11] Garakani MA, Abouali S, Zhang B, Xu ZL, Huang J, Huang JQ (2015). Controlled synthesis of cobalt carbonate/graphene composites with excellent supercapacitive performance and pseudocapacitive characteristics. J Mater Chem A.

[12] Wang H, Zhang L, Tan X, Holt CMB, Zahiri B, Olsen BC (2011). Supercapacitive properties of hydrothermally synthesized Co_3_O_4_ nanostructures. J Phys Chem C.

[13] Li Y, Zhao N, Shi C, Liu E, He C (2012). Improve the supercapacity performance of MnO_2_-decorated graphene by controlling the oxidization extent of graphene. J Phys Chem C.

[14] Yang W, Gao Z, Ma J, Wang J, Wang B, Liu L (2013). Effects of solvent on the morphology of nanostructured Co_3_O_4_ and its application for high-performance supercapacitors. Electrochim Acta.

[15] Kumar R, Kim HJ, Park S, Srivastava A, Oh IK (2014). Graphene-wrapped and cobalt oxide-intercalated hybrid for extremely durable super-capacitor with ultrahigh energy and power densities. Carbon.

[16] Liao Q, Li N, Jin S, Yang G, Wang C (2015). All-solid-state symmetric supercapacitor based on Co_3_O_4_ nanoparticles on vertically aligned graphene. ACS Nano.

[17] Guan Q, Cheng J, Wang B, Ni W, Gu G, Li X (2014). Needle-like Co_3_O_4_ anchored on the graphene with enhanced electrochemical performance for aqueous supercapacitors. ACS Appl Mater Interfaces.

[18] Huang ML, Gu CD, Ge X, Wang XL, Tu JP (2014). NiO nanoflakes grown on porous graphene frameworks as advanced electrochemical pseudocapacitor materials. J Power Sources.

[19] Zhao G, Li J, Ren X, Chen C, Wang X (2011). Few-layered graphene oxide nanosheets as superior sorbents for heavy metal ion pollution management. Environ Sci Technol.

[20] Li X, Song Q, Hao L, Zhi L (2014). Graphenal polymers for energy storage. Small.

[21] Jiang Y, Li J, Hao JSuzhou Graphene-Tech Co., Ltd. Chinese Patent. 201410244717.0.

[22] Meher SK, Rao GR (2011). Ultralayered Co_3_O_4_ for high-performance supercapacitor applications. J Phys Chem C.

[23] Xiang C, Li M, Zhi M, Manivannan A, Wu N (2013). A reduced graphene oxide/Co_3_O_4_ composite for supercapacitor electrode. J Power Sources.

[24] Liu X, Men C, Zhang X, Li Q (2016). An extraordinary sulfonated-graphenal-polymer-based electrolyte separator for all-solid-state supercapacitors. Small.

[25] Deori K, Deka S (2013). Morphology oriented surfactant dependent CoO and reaction time dependent Co_3_O_4_ nanocrystals from single synthesis method and their optical and magnetic properties. CrystEngComm.

[26] Ujjain SK, Singh G, Sharma RK (2015). Co_3_O_4_@reduced graphene oxide nanoribbon for high performance asymmetric supercapacitor. Electrochim Acta.

[27] Wu ZS, Zhou G, Yin LC, Ren W, Li F, Cheng HM (2012). Graphene/metal oxide composite electrode materials for energy storage. Nano Energy.

[28] Sun CY, Zhu YG, Zhu TJ, Xie J, Cao GS, Zhao XB (2013). Co(OH)_2_/graphene sheet-on-sheet hybrid as high-performance electrochemical pseudocapacitor electrodes. J Solid State Electrochem.

[29] Yang Q, Lu Z, Chang Z, Zhu W, Sun J, Liu J (2012). Hierarchical Co_3_O_4_ nanosheet@nanowire arrays with enhanced pseudocapacitive performance. RSC Adv.

[30] Zhu YG, Wang Y, Shi Y, Wong JI, Yang HY (2014). CoO nanoflowers woven by CNT network for high energy density flexible micro-supercapacitor. Nano Energy.

[31] Dong Y, He K, Yin L, Zhang A (2007). A facile route to controlled synthesis of Co_3_O_4_ nanoparticles and their environmental catalytic properties. Nanotechnology.

[32] Liang Y, Li Y, Wang H, Zhou J, Wang J, Regier T (2011). Co_3_O_4_ nanocrystals on graphene as a synergistic catalyst for oxygen reduction reaction. Nat Mater.

[33] Feng C, Zhang J, He Y, Zhong C, Hu W, Liu L (2015). Sub-3 nm Co_3_O_4_ Nanofilms with Enhanced Supercapacitor Properties. ACS Nano.

[34] Chang MS, Kim T, Kang JH, Park J, Park CR (2015). The effect of surface characteristics of reduced graphene oxide on the performance of a pseudocapacitor. 2D Mater.

[35] Li T, Li S, Zhang B, Wang B, Nie D, Chen Z (2015). Supercapacitor electrode with a homogeneously Co_3_O_4_-coated multiwalled carbon nanotube for a high capacitance. Nanoscale Res Lett.

[36] Lu X, Zheng D, Zhai T, Liu Z, Huang Y, Xie S (2011). Facile synthesis of large-area manganese oxide nanorod arrays as a high-performance electrochemical supercapacitor. Energy Environ Sci.

